# Enhancing Cyber-Resilience for Small and Medium-Sized Organizations with Prescriptive Malware Analysis, Detection and Response

**DOI:** 10.3390/s23156757

**Published:** 2023-07-28

**Authors:** Lucian Florin Ilca, Ogruţan Petre Lucian, Titus Constantin Balan

**Affiliations:** Faculty of Electrical Engineering and Computer Science, “Transilvania” University of Brasov, 500036 Brasov, Romania; lucian.ilca@unitbv.ro

**Keywords:** polymorphic malware, zero-day exploits, rootkits, botnets, ransomware, APTs, fileless malware, sandbox evasion, obfuscation, C&C, memory-based attacks, MaaS, exploit kits, AETs, advanced malware analysis, prescriptive, SMEs

## Abstract

In this study, the methodology of cyber-resilience in small and medium-sized organizations (SMEs) is investigated, and a comprehensive solution utilizing prescriptive malware analysis, detection and response using open-source solutions is proposed for detecting new emerging threats. By leveraging open-source solutions and software, a system specifically designed for SMEs with up to 250 employees is developed, focusing on the detection of new threats. Through extensive testing and validation, as well as efficient algorithms and techniques for anomaly detection, safety, and security, the effectiveness of the approach in enhancing SMEs’ cyber-defense capabilities and bolstering their overall cyber-resilience is demonstrated. The findings highlight the practicality and scalability of utilizing open-source resources to address the unique cybersecurity challenges faced by SMEs. The proposed system combines advanced malware analysis techniques with real-time threat intelligence feeds to identify and analyze malicious activities within SME networks. By employing machine-learning algorithms and behavior-based analysis, the system can effectively detect and classify sophisticated malware strains, including those previously unseen. To evaluate the system’s effectiveness, extensive testing and validation were conducted using real-world datasets and scenarios. The results demonstrate significant improvements in malware detection rates, with the system successfully identifying emerging threats that traditional security measures often miss. The proposed system represents a practical and scalable solution using containerized applications that can be readily deployed by SMEs seeking to enhance their cyber-defense capabilities.

## 1. Introduction

In a constantly evolving cyber environment, the procedures, processes, and methodologies for security incident response play a vital role in ensuring IT security in modern institutions. To counter new types of attacks and keep up with the rapid pace of changes in the cyber landscape, it is essential to adapt and develop effective strategies. This research work focuses on the implementation of well-defined systems, procedures, and processes that enable institutions to respond to security incidents promptly and efficiently. Within this context, the emphasis lies on the identification and management of emerging attack vectors, with particular attention given to malicious binaries. These attacks pose an increasingly significant threat as cyber adversaries become more sophisticated and employ subtle techniques to evade detection [1].

To address these challenges, institutions must be prepared to rapidly detect and respond to cyber-attacks. The implementation of advanced methodologies and the development of analytical and investigative capabilities are key factors in establishing a robust defense against attacks of any nature. The utilization of cutting-edge technologies, such as artificial intelligence and behavioral analysis [2] can enhance the effectiveness of security efforts and provide a deeper understanding of attack patterns and adversary behavior.

This article includes a novel proposed solution and methodologies designed specifically for detecting new emerging threats. The solution leverages open-source technologies, implements a Dockerized infrastructure, and employs fully automated processes to cater to small and medium-sized enterprises (SMEs) with up to 250 endpoints.

The proposed solution incorporates a comprehensive range of integrated open-source solutions, including XDR/EDR, AV Sandbox, SIEM, improved Firewall, NIPS, HIDS, Infrastructure Monitoring, MDM, etc. These components collectively contribute to enhancing malware detection capabilities and incident response efficiency. Specifically, the solution addresses challenges related to malware detection and incident response, such as DLL injection techniques [3] employing mutation queries and process hollowing; the use of AI to develop mutating malware that evades detection, overcoming class imbalance problems in classification and detection; and other challenges, which are detailed in Chapter 3 of this research.

It is important to note that the proposed solution does not function as a standalone antivirus solution but rather operates as an open-source security infrastructure. Its primary purpose is to detect new malware families and internal threat attacks and serve as a prescriptive SOC (Security Operations Centre) for SMEs. The solution encompasses extended capabilities for malware detection, threat intelligence sharing, network protection, and security awareness.

The research study showcases the tools employed in building the proposed solution and includes two test cases that demonstrate the solution’s efficacy using different testing methods. Additionally, a comparative study is conducted to evaluate the performance of the proposed solution in comparison to other antivirus engines.

It is worth highlighting that the proposed solution offers SMEs an affordable and adaptable security infrastructure, enabling them to proactively detect and respond to evolving threats. The solution’s key features include its reliance on open-source technologies, the utilization of Docker for seamless deployment, and its emphasis on automation to streamline security operations. By leveraging these capabilities, SMEs can fortify their defenses against new malware strains and internal threats and benefit from enhanced threat intelligence sharing, network protection, and security awareness.

## 2. Organization Security Objectives and Policies

Starting from the information security policy that defines the organization’s objectives regarding information security and establishes the basic rules and principles that employees must follow, security procedures provide detailed guidance on how to implement security policies and rules. Standards set the minimum-security requirements that must be adhered to, while security guidelines provide recommendations and best practices for ensuring a safe working environment. Business continuity plans are developed to ensure the continuity of activities in the event of incidents or emergencies. A detailed description will be provided for the procedures, policies, and technical and practical implementations regarding two fundamental aspects of information security: security incident management policy and network security policy.

The several key differentiators and innovations that set it apart from other solutions will be the following:

Open-source and free: The entire solution is open-source and free, providing a cost-effective alternative to other solutions in the market. This can be useful for organizations with budget constraints.

Easy deployment using containerization: Leveraging containerized application development with Docker simplifies the deployment process. With a custom build script, organizations can choose to deploy the entire solution or specific components based on their requirements. This flexibility makes it easier for businesses to adopt and customize the solution according to their needs.

Backup for enterprise security solutions: The solution can act as a backup or alternative in case a company’s existing enterprise solution encounters issues or downtime. This provides additional security and peace of mind for organizations relying on top-notch enterprise solutions [4].

Quick deployment for SMEs: With the ability to deploy and install the solution in just one day, this solution could help small and medium-sized enterprises (SMEs) with a rapid implementation process. This reduces the time and effort required to integrate the solution into the infrastructure, making it more accessible for SMEs with limited resources.

User-friendly interface and quick learning curve: The solution’s user interface is designed to be intuitive and easy to use, allowing the IT department personnel at SMEs to quickly become proficient within a day. This reduces the training time and ensures that organizations can start utilizing the solution.

Scalability for various infrastructures: The solution scalability feature allows for the design and preparation to accommodate different infrastructure sizes and environments. This flexibility ensures that the solution can be adapted to every one of the organization’s needs, whether it is a small-scale deployment or a larger enterprise infrastructure.

The main security policies [5] aimed at risk reduction are essential in ensuring a secure and protected environment within institutions. These policies constitute a set of rules, directives, and procedures that are implemented to identify, assess, and manage security risks and prevent unwanted incidents. There are several security policies [5] to ensure the confidentiality, integrity, and availability of data:

(I) General Security Policy—The primary document that defines the objectives, principles, and responsibilities in the field of information security within the organization. It should provide an overview of the approach and security strategy.

(II) Information Resources Usage Policy—The document that establishes rules and restrictions regarding the use of the organization’s information resources, including equipment, networks, software, and data.

(III) Information Protection Policy—The document that defines the necessary measures and procedures to protect the organization’s sensitive and confidential information, including encryption, access management, and activity monitoring.

(IV) Password Management Policy—The document that establishes rules and practices for creating, using, and managing passwords within the organization, including requirements for password complexity, change frequency, and the use of multi-factor authentication.

(V) Physical Security Policy—The document that describes the security measures taken to protect the organization’s physical infrastructure, including access control to premises, video monitoring, fire protection, and natural disaster prevention.

(VI) Network Security Policy—The document that establishes measures and rules for protecting the organization’s network, including firewall configuration, intrusion detection and prevention, traffic management, and security event monitoring.

(VII) Security Incident Management Policy—The document that describes the procedures and responsibilities for managing security incidents, including incident reporting, investigation, remediation, and communication of incidents to stakeholders (other institutions).

(VIII) Training and Awareness in Security Policy—The document that establishes the requirements and program for training and raising awareness among employees in terms of information security to educate and prepare them regarding threats and security practices.

This research paper proposes a focus on technical implementation using two organization-level policies: Security Incident Management Policy and Network Security Policy.

(a) The Security Incident Management Policy [6] aims to ensure a prompt and efficient response to security incidents. This involves implementing a set of well-defined procedures and processes that enable the identification, evaluation, and management of security incidents in a proper manner. Through this policy, the institution prepares itself to face cyber threats and minimize their impact. The Security Incident Management Policy includes stages such as incident detection, impact assessment, isolation and remediation of the incident, investigation of causes, and the implementation of preventive measures to avoid similar incidents in the future.

(b) The Network Security Policy [7] is responsible for ensuring a secure and protected environment within the institution’s network infrastructure. This entails implementing a set of rules and controls to prevent unauthorized access, protect data, and ensure the confidentiality and integrity of information transmitted through the network. The Network Security Policy includes aspects such as network segmentation, user authentication and authorization, network traffic monitoring, equipment management, and regular updates of security systems. By implementing this policy, the institution ensures the protection of the network and information, minimizing the risks of cyber-attacks and data loss.

**Figure A1:**
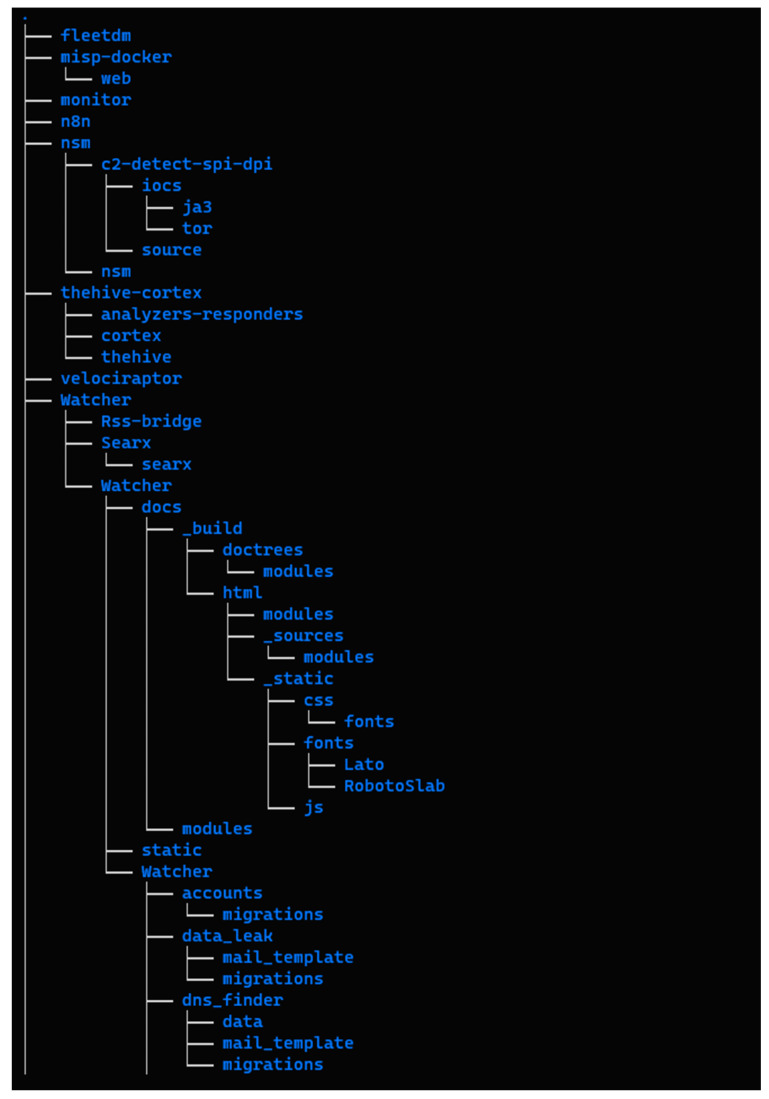
Directory tree of the proposed solution, containing all the files, directories, and applications (1).

**Figure A2:**
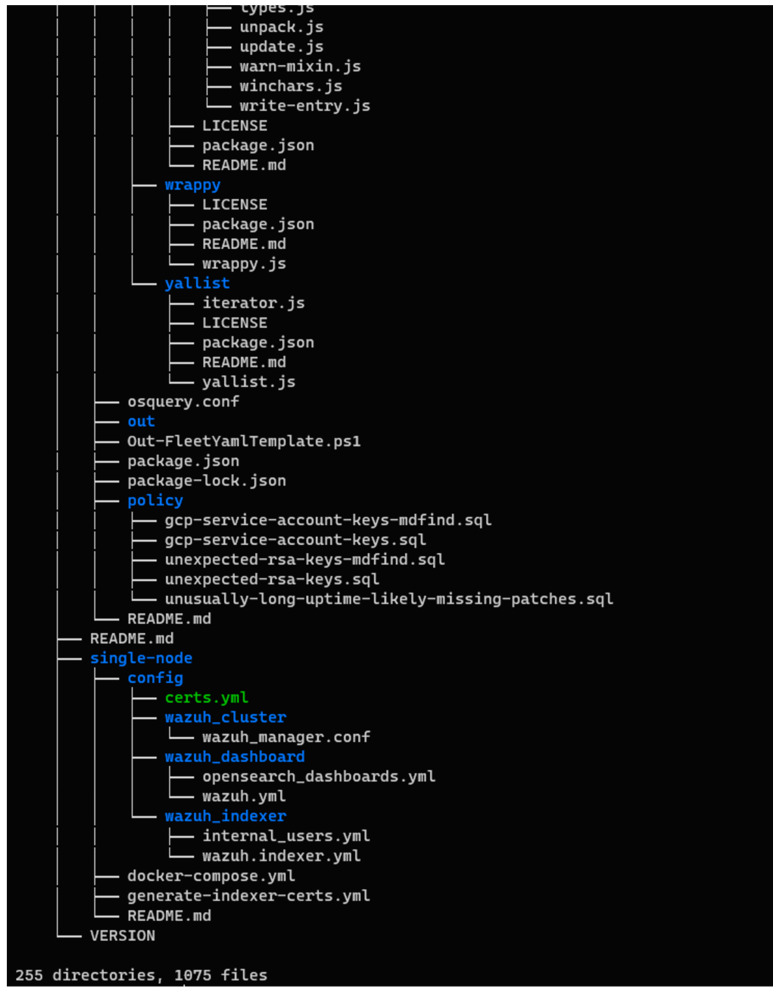
Directory tree of the proposed solution (2).

**Figure A3:**
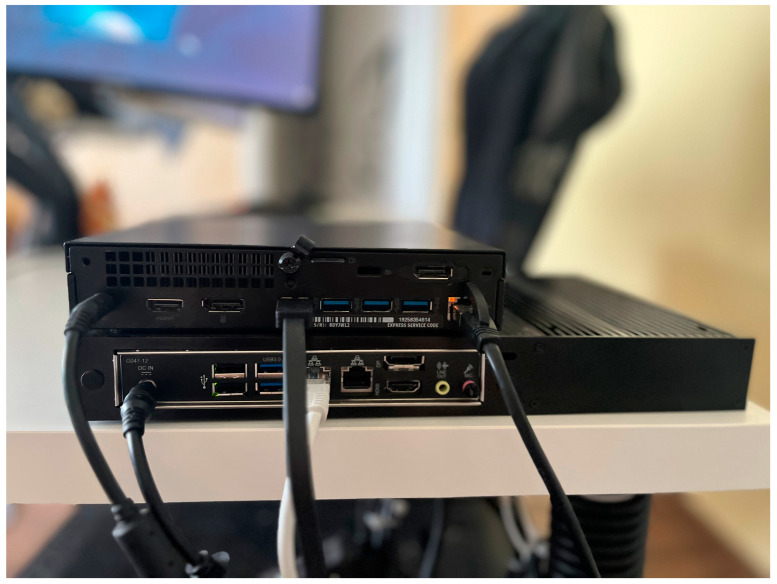
Infrastructure components (hardware) used for building the security stack solution.

Starting from the essential aspects of network security policy and incident management and response, this research provides a detailed analysis. The primary objective of this study is to thoroughly examine the network security policy and the policy for incident management and response to develop a comprehensive understanding of critical factors contributing to its technical success and effectiveness through practical implementation using open-source systems capable of observation, detection, and response. Concepts such as asset protection, prevention of unauthorized access, ensuring data confidentiality and integrity, and efficient response to security alerts and issues are explored using technical language and specific terminology. Additionally, the implementation of advanced network protection methods against increasingly sophisticated cyber threats is demonstrated. Technological solutions and innovative strategies, including the use of firewalls, intrusion detection systems, data encryption, and network traffic monitoring, are analyzed. By implementing and coordinating these technologies and systems, as well as integrating them into network security policies and incident response, the aim is to develop efficient solutions tailored to the specific needs of small and medium-sized institutions/companies with up to 250 individuals.

(I) Multi-module firewall [8]—A multifunctional firewall can be an essential component of the network security policy, providing traffic filtering and protecting the network against unauthorized access. The policy should establish rules and configurations for the firewall, including permissions and restrictions regarding network traffic.

(II) SIEM [8]—Optimizing management, analysis, and information security event strategies through the implementation of advanced logging and monitoring solutions.

(III) Extended Detection and Response (XDR) system/EDR (Detection and Response for Endpoints)—The extended detection and response (XDR) and (EDR) detection and response for endpoints solutions with extended system response functionality can represent a major component in the network security policy, offering advanced threat detection and incident response. The policy should include requirements and procedures for implementing and utilizing the solution, including monitoring and alerting for security events.

(IV) Network Security Module (NSM)—Integrating the NSM solution as a network security module is part of the network security policy, providing advanced protection against cyber threats and attacks. The policy should define the configurations and usage policy of the solution or current system, including the implementation and monitoring of specific functionalities.

(V) Network/System Intrusion Prevention System (IPS)—The intrusion prevention system (IPS) can be included in the Network Security Policy, providing detection and active blocking of known attacks and suspicious behaviors on the network. The policy should cover the implementation and configuration of the system, as well as defining rules and specific incident response actions.

(VI) Security Incident Response and Anti-Malware Sandbox Module—This module focuses on promptly and effectively responding to security incidents and testing the efficacy of malware detection and response.

(VII) Integration of Identity and Access Management Solution—This integration ensures that only authorized individuals have access to critical resources, reducing the risk of unauthorized access and potential security breaches.

(VIII) Integration of Threat Hunting Solution—The integration with existing security systems and tools provides comprehensive visibility into network activity, allowing for the early detection of suspicious patterns or indicators of compromise.

(IX) Integration of Digital Forensics and Incident Response module—This module combines the principles of digital forensics and incident response to streamline the process of identifying, containing, and analyzing potential security breaches.

(X) Security Orchestration, Automation, and Response (SOAR) module—This module combines advanced technologies, intelligent automation, and streamlined workflows to enhance the efficiency and effectiveness of security operations. Other information related to the proposed solution, services, number of files and directories related to the security solution could be found on the Appendix, on Figure A1, Figure A2 and Figure A3.

The original contribution of this research lies in an innovative approach to network security and incident response by developing advanced integrations between different open-source solutions and enhancing them, along with the development of a Network Security Module (NSM). A distinctive feature of this contribution is the ability to rapidly deploy and implement the proposed solutions with minimal processing resources required. This efficient and easy-to-implement approach provides an accessible way to utilize the mentioned solutions without complex configurations or extensive technical efforts. By adopting this method, institutions or organizations can benefit from protecting their infrastructure and critical data, ensuring the secure implementation and utilization of network security policies.

Security incident management [8] constitutes a crucial component of an organization’s cybersecurity strategy. It establishes the necessary structure and methodologies for identifying, assessing, reporting, and resolving security incidents that could potentially impact the IT infrastructure and operations of the entity. A well-defined strategy for security incident management aims to mitigate the negative impact of incidents and ensure an effective response to them. It includes defining responsibilities and communication procedures, both within the organization and in coordination with relevant authorities and other entities [9].

A key component of the security incident management policy is the establishment of an incident classification and prioritization system based on the severity and impact of incidents on the entity. This facilitates efficient resource allocation and appropriate incident management according to their relevance. The security incident management strategy also includes provisions for notifying and reporting incidents to relevant stakeholders, such as authorized authorities, clients, business partners, or other relevant organizations. This approach aims to efficiently implement cybersecurity, ensuring operational continuity and protecting the organization’s essential data and information.

This research presents a series of fundamental and innovative achievements in the field of cybersecurity that redefine standards for information protection and management. Open-source solutions are used exclusively to minimize costs, and containerized applications are utilized for scalability. The following are just a few relevant contributions:

Establishing a security incident response platform by creating a system capable of swiftly and efficiently responding to potential threats and security incidents.

Organizing security information and events and highlighting the importance of collecting, analyzing, and understanding information related to information security to facilitate informed decision-making.

To calculate the number of security incidents occurring in a system over time, a differential equation will be used.

*y* = the number of security incidents, reflected by dynamic nature of incidents within a system, capturing the fluctuations and transformations over time.

*P* = the system’s incident management capacity.

*k* = the rate of growth of security incidents.

*dy*/*dt* = rate of change of the variable *y* with respect to time, unveiling the temporal evolution aspect.

*P* = the incident management capacity of the system.

*k* = the rate of growth of security incidents, quantifying the impact and magnitude of the factors.

1 − *y*/*P* = balance between the occurrence of incidents and the maximum capacity of the system.

This approach enables the quantitative analysis and prediction of security incidents within a system, allowing for a deeper understanding of the system’s vulnerability and the effectiveness of its incident management capabilities. By modeling the relationship between incident count, incident management capacity, and growth rate, organizations can make informed decisions regarding resource allocation, risk mitigation strategies, and the improvement of incident response processes.

The differential equation serves as a mathematical representation of the dynamic nature of security incidents, considering the interplay between incident management capacity and the ongoing evolution of security threats. By monitoring and adjusting the variables in the equation, organizations can optimize their incident response efforts, ensuring a proactive and effective approach to addressing security incidents. This equation encapsulates the dynamic interplay between the occurrence of security breaches and the system’s ability to handle and mitigate them.

$$\frac{dy}{dt}=k\cdot y\cdot \left(1-\frac{y}{P}\right)$$

A differential equation with separated variables is being solved by applying the method of integration to find the general solution of the equation, which takes the form:

$$\int_{y}^{1}+\frac{1}{P-y}\;dy=\int k\;dt$$

where *y* and *t* represent the independent variables, and *P* and *k* are known constants. The integral on the left-hand side represents the sum of integrals of two distinct functions of *y*, while the integral on the right-hand side represents the integral of a constant function of *t*. By finding the antiderivatives of these functions and applying the corresponding initial conditions, the solution of the differential equation can be determined.

Applying the technique of partial fractions will begin to disentangle the intricate composition [10] of the terms. The integral symbol (∫) serves as a gateway to the realm of calculus, guiding our path towards the evaluation of an infinite series. Within this mathematical framework, it will result in the functions 1/*y* and 1/(*P* − *y*), representing the objects of the integration endeavors in the current context. The variable *y* captures the current state of security incidents within the system under examination. Its fluctuations mirror the ever-changing landscape of threats, encapsulating the dynamic and evolving environment. In parallel, *P* emerges as a sentinel, guarding the system’s boundaries against the encroachment of incidents. It represents the theoretical upper limit, the threshold beyond which the system’s capacity for incident management may be stretched.

*dy* denotes the change in the number of incidents, unraveling the intricacies of security breaches. Simultaneously, *dt*, the herald of temporal dynamics, endows a profound understanding of the relationship between time and incidents, encapsulating the underlying dynamics of the system. The constant, *k*, shows the path of growth or decline in the number of security incidents. It serves as a quantitative measure reflecting the rate at which these incidents multiply or diminish. By comprehending this constant of proportionality, it will reveal a new and deeper understanding of the system’s behavior, paving the way for insightful analyses and effective mitigation strategies.

The natural logarithms ln|*y*| and ln|*P* − *y*|, which serve as exquisite metrics, measure the temporal trajectory required for the number of incidents, *y*, to reach a certain level, as well as the time needed for the system to approach *P* − *y*, the remaining incidents until the critical threshold, *P*, is reached.

The resultant integration of the right-hand side of the differential equation yields *kt*, a part to the integration process wherein *k*, a proportionality constant, assumes its state, and *t* emerges as part of the time itself.

Moreover, the constant *C* enters the stage using the role as a constant of integration, a reflection of the initial conditions that govern the problem at hand. Its precise value becomes contingent upon the specifications of the initial conditions.

The expressions ln|*y*| and ln|*P* − *y*| = *kt* + *C* unveil the interplay between the recorded incidents, (*y*); the temporal dimension, (*t*); and the growth rate, (*k*). They encapsulate the balance between time, incidents, and the system’s inherent dynamics.

ln|y|−|ln|P−y|=kt+C

As in previous examples demonstrating this sigmoid function, the following equation allows for finishing the calculation for the value of *y* at different time points based on these parameters:

$$y=\frac{P}{1+e\;{-}^{kt-C}}$$

This equation represents the number of incidents, denoted by *y*, as a function of time; growth rate, (*k*); integration constant, (*C*); and incident threshold, (*P*). The logistic equation describes how the number of incidents initially grows rapidly but slows down as it approaches the maximum incident threshold. This reflects a common characteristic of many real-world systems [11], where the initial rapid growth is constrained by limitations or maximum capacities.

**Figure 1:**
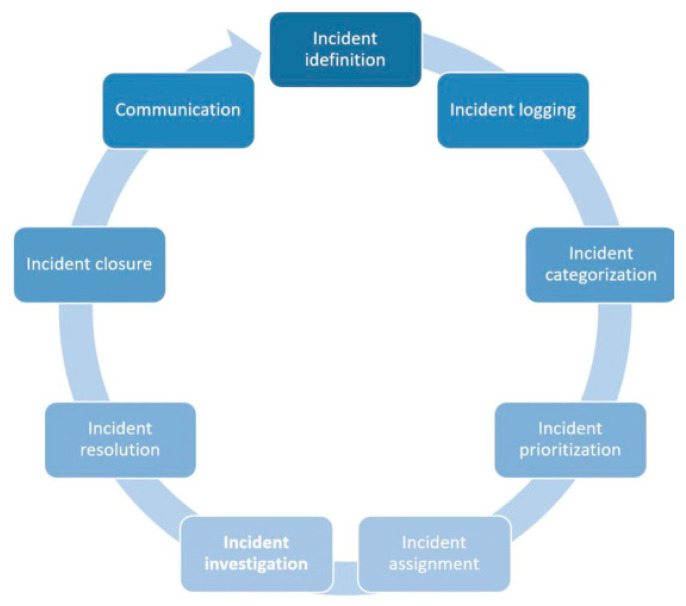
Incident Management Flow.

As shown in Figure 1, the incident management flow illustrates the systematic process of handling incidents. The workflow encompasses various stages, beginning with incident identification and concluding with incident closure and communication.

## 3. Difficulties in Malware Detection and Incident Response

The detection of malware and the effective response to security incidents pose significant challenges [12]. With the ever-evolving threat landscape and the increasing sophistication of malicious actors, organizations struggle to keep up with the detection and mitigation of malware attacks. Malware detection involves the identification and analysis of malicious software that can compromise the security and integrity of systems and networks.

Traditional signature-based detection methods [12] are often inadequate in detecting emerging and polymorphic malware variants [13], leading to a significant time gap between malware release and detection. This time gap allows malware to spread and cause damage before countermeasures can be implemented. Moreover, incident response, which encompasses the steps taken to investigate, contain, eradicate, and recover from security incidents, is a complex and multifaceted process. It requires skilled professionals, well-defined procedures, and effective collaboration across various teams and stakeholders. However, organizations often face challenges in coordinating incident response efforts, accurately assessing the impact and scope of incidents, and implementing timely remediation measures.

A. DLL injection techniques using mutation queries and process hollowing [14]

In the realm of malware detection, one of the key challenges is bypassing antivirus (AV) systems to execute malicious code. Leveraging LLVM and AI as code generation tools introduces a unique dilemma: ensuring the functional validity of the obtained code. While AI tools can generate malicious code, they lack the ability to test its functionality, placing the responsibility on the malware’s developers to validate the code and ensure its proper execution. To shed light on the validation process, a file encryption scenario was used. For the malware to successfully encrypt files, it must validate several critical actions: reading a file, encrypting it, writing the encrypted file to the filesystem, and decrypting it accurately.

A potential approach to address this challenge involves the following steps:

The malware establishes communication with a command and control (C&C) server, requesting a file encryption function from an AI cloud-based tool.

Upon receiving the code in text form, the malware generates a test file with known content and encrypts it using the specified encryption key. The malware transmits the encrypted test file back to the C&C server, which attempts to decrypt it using the same key. The C&C server performs validation checks on the decrypted file. If successful, it instructs the malware to proceed with encrypting the desired files. Otherwise, the process is repeated until a valid encryption function is obtained. This method allows for iterative refinement of the code obtained from the AI, ensuring that the malware can effectively execute the desired actions while evading AV detection. By validating the functionality of the generated code through test scenarios, developers can enhance the efficiency and success rate of AV bypass techniques.

B. Using AI for development-mutating malware that evades detection [15]

Recent advancements in generative AI have led to the development of proof-of-concept models for polymorphic malware. These models demonstrate the potential of dynamically mutating malware payloads to evade detection and bypass endpoint and response (EDR) filters. One such proof of concept, called BlackMamba [16], utilizes the ChatGPT API to prompt and generate a polymorphic keylogger payload in Python. By leveraging Python’s exec() function, the payload mutates at runtime, making it challenging to detect. BlackMamba successfully evaded an “industry-leading” EDR application in multiple instances, showcasing its effectiveness. Another proof of concept, ChattyCat, developed by cybersecurity company CyberArk, incorporates ChatGPT [16] directly into the malware. The malware periodically queries ChatGPT for new modules that execute malicious actions. This approach allows for easy acquisition of a new code or modification of an existing code, providing a template to build various types of malware, including ransomware and info stealers. These proofs of concept highlight the creative use of generative AI in the development of polymorphic malware. They also shed light on certain weaknesses in content filters when using the ChatGPT API, which is not as stringent as the online version. This poses challenges for regulatory efforts, as the technology industry is still exploring the full extent of generative AI’s capabilities and determining appropriate regulations to address potential harm.

C. Overcoming class imbalance problem in classification and detection [17]

The application of machine-learning techniques in practical domains has sparked the examination of imbalanced datasets and their impact on classification performance. Imbalance refers to the uneven distribution of classes within existing datasets, where certain classes have significantly more instances than others, resulting in a skewed representation. For instance, in a population sample, differentiating between twin-birth and singleton-birth individuals would yield a greater number of singleton-birth examples. This class imbalance is inherent in many classification problems, although it can also be an artifact of the dataset creation process, such as sampling limitations. Addressing the class imbalance problem can be approached from two levels: data and algorithmic [18]. At the data level, various resampling techniques can be employed, such as over-sampling, under-sampling, and directed or undirected sampling approaches. Algorithmically, adjustments can be made to class costs, probabilistic estimates, and decision thresholds. Additionally, single-class learning can be explored as an alternative to traditional two- or multi-class learning methods. It is also important to consider within-class imbalance, where sub-clusters may exist within a class but have unequal distributions. Previous studies have investigated the effects of malicious file percentage (MFP) in training sets. However, defining a realistic MFP is challenging, as it involves approximations based on previous findings and the nature of the dataset. The representation of real-life distributions remains a subject of debate and depends on various factors and user scenarios.

D. One-class Support Vector Machines (SVMs) for imbalanced datasets [19]

In certain scenarios, Support Vector Machines (SVMs) applied to imbalanced datasets can be enhanced by employing single-class learning instead of traditional two-class learning approaches. SVMs can handle large numbers of negative instances (majority class) that are far from the decision boundary, as they primarily rely on the instances close to the boundary (Support Vectors) for classification. One-class learning offers the advantage of training the model on a single class, which can alleviate the concern of malicious file percentage (MFP) size. However, within-class imbalances can still have an impact on performance. In a study by Santos et al., one-class learning was applied to a statically generated dataset of run-time opcode traces to determine the better detection performance and the required number of labeled samples for accuracy. The authors used the ROC–SVM algorithm, which combines SVM with the Rocchio method, and selected significant negative instances to generate multiple classifiers iteratively. The top 1000 features were selected using Information Gain for training the SVM model. The results showed that as the number of labeled instances increased, the overall performance in malware labeling improved, but the recall (true positive detection rate) decreased. The accuracy achieved was 83.432% when 600 out of 1000 malware samples were labeled, and precision increased with the size of the labeled set. However, the precision for legitimate samples decreased as the number of labeled malware instances increased, resulting in more false negatives. The authors obtained an accuracy and F-measure of over 85% when the training set contained 60% labeled data from benign ware, but performance declined after 600 labeled instances. It is important to note that the dataset used in this study was statically generated and did not account for packed malware. Additionally, the malware dataset composition varied in terms of malware types and the number of instances per type. The application of one-class learning can mitigate the need for a representative MFP level and simplify the model to focus on a single category.

E. A comparative analysis of classifiers for malware detection in dynamic environments [4]

Kang et al. (2014) conducted a comparative study of five classifiers, including the Bayesian network, sequential minimal optimization algorithm (SMO), K-nearest-neighbor (KNN), repeated incremental pruning to produce error reduction (RIPPER), and Random Forests. Among these, Random Forests exhibited the highest accuracy in terms of true positive (TP) detection, precision (p), recall (r), and F-measure (f) while also demonstrating the lowest false positive (FP) rate and testing time (tet). Although the training time (trt) of Random Forests was surpassed by KNN, it remained significantly faster than SMO, which showed the poorest performance across all metrics. Another ensemble method employed by Menahem et al. (2009) [20] combined five base-inducing classifiers (C4.5, Naïve Bayes, KNN, VFI, and OneR) for malware detection. The researchers utilized an n-gram analysis of binary representations with varying n values (ranging from three to six), along with PE features and function-based features. However, no specific justification was provided for the choice of n-gram size. The Troika ensemble method was identified as the most accurate, especially when dealing with multi-class datasets. The authors cautioned against the use of Bayes ensemble methods due to their low accuracy and Area Under the Curve (AUC) measurements. Notably, there is a scarcity of literature exploring the cross-comparison of classifiers using dynamically derived opcodes, suggesting a potential avenue for further investigation.

F. Unknown malware types (Example: Ransomware) [21]

Ransomware is a malicious software that aims to generate illicit profits through social engineering tactics, originating from the family of fake antivirus malware. It operates by holding a device hostage, either by locking the machine or encrypting its data, and demanding a ransom, typically around USD 300, for its release. Two primary categories of ransomware can be observed: Locker, which restricts access to the device except for the payment interface, and Crypto, which denies access to files and data. To ensure payment, ransomware perpetrators often employ social engineering techniques such as implanting illicit content, posing as law enforcement, or damaging the reputation of businesses. With the advent of the Internet of Things (IoT), the potential for ransomware to infiltrate various aspects of our daily lives is significant. The FBI estimated that criminals earned over USD 1 billion from ransomware in 2016 [22], highlighting the ongoing rise of this threat. Despite the increasing prevalence of ransomware, there is a lack of research focused on the dynamic analysis of runtime traces specifically for this type of malware. Applying classification methods, as previously discussed, may provide an opportunity to develop anti-ransomware systems capable of mitigating the growing risks associated with these malware variants.

Obfuscation techniques [3] pose significant challenges for malware detection, affecting both antivirus vendors and researchers. Previous efforts in malware research have been hindered using evasion strategies employed by malware authors. Machine-learning algorithms have been explored for classifying malware based on previously unseen datasets. Schultz et al. (2001) [23] conducted an influential study in this domain, comparing three classifiers against a signature-based commercial AV-scanner. Various features, such as program headers, string features, and byte sequences, were examined. The classifiers exhibited superior accuracy compared to the scanner, doubling its performance. In another study by Kolter and Maloof, Boolean n-gram analysis was applied to the hexadecimal representation of malware executables [24], yielding excellent accuracy using a boosted decision tree classifier with an area under the receiver operating characteristic (ROC) curve of 0.996. Moskovitch et al. [25] collected data on 323 features per second across five unseen worm variants and achieved an average detection rate of over 90% accuracy using only 20 features, with certain worm variants surpassing 99% accuracy.

G. Hidden Markov Models [26]

Anderson et al. (2011) [27] conducted research on dynamically yielded run-time traces represented by weighted directed graphs modeled as Markov chains. Their objective was to demonstrate the superior performance of their method compared to n-gram representations and signature-based detection algorithms. The dataset consisted of 1615 malware samples and 615 benign samples, but the authors did not provide detailed information regarding malware type, family, age, obfuscation, or size, limiting the interpretation of their analyses. The Markov model achieved an accuracy of 96.41%, with 47 false positives (FPs) and 33 false negatives (FNs). However, the authors noted that the FN rating might be an artifact of the sampling due to the dataset’s positive class skew, emphasizing the issue of data collection.

In the study by Lin and Stamp (2011) [28], weaknesses in Hidden Markov Model (HMM)-based detection tools susceptible to metamorphic malware were explored. The authors developed a kit for creating metamorphic viruses and successfully evaded detection by HMM algorithms using the derived malware samples. They found that high levels of metamorphism alone were not sufficient for evasion; instead, altering the code to resemble benign ware through the injection of subroutines from normal files led to a rise in misdetection.

Attaluri et al. (2009) [29] investigated metamorphic malware using Profile HMMs (PHMMs) and code slicing with jumps as an obfuscation tactic. A reduced instruction set of 36 opcodes, along with a wildcard for all other opcodes, was employed to detect malware generated by three separate metamorphic kits. The sample sizes were small (10, 30, and 200), and a high error rate was observed for the largest class, rendering the solution impractical.

Vemparala et al. (2016) [30] compared traditional HMMs with PHMMs based on the frequency of the 36 most common API calls using both static and dynamic analysis. Dynamic analysis outperformed static analysis for all but one of the seven examined malware types when utilizing both regular HMM and PHMM (which were only obtained dynamically).

Thunga and Neelisetti (2015) [31] trained HMMs to identify three families to which 1500 malicious samples belonged. Their models achieved success rates ranging from 88.4% to 90.86%, surpassing two major AV products and the work of Austin et al. (2013) [32]. However, this research did not aim to detect malware from benign ware but rather confirmed the accepted family label assigned by Nappa et al. (2013), upon which the models of Thunga and Neelisetti (2015) relied for accuracy.

H. Supervised vs. Unsupervised ML algorithms APT detection

Difficulties arise in the field of malware detection due to the complex nature of the problem. One of the challenges lies in accurately classifying malware samples amidst a vast and ever-evolving landscape of threats. Classification methods, such as Support Vector Machines, decision trees, and Random Forests, are utilized to differentiate between malware and benign software. However, the diversity and polymorphic nature of malware make it difficult to create comprehensive and effective classification models.

Another challenge in malware detection is the ability to predict numerical values related to malware behavior. Regression analysis techniques, such as linear regression, logistic regression, and polynomial regression, can help estimate variables such as revenue loss or potential damage caused by a specific malware strain. However, obtaining reliable and comprehensive data for accurate regression models can be challenging due to the constantly evolving nature of malware and its impact. Clustering techniques present yet another challenge. Clustering algorithms, such as K-means clustering, can be applied to similar groups of malware samples based on their characteristics. However, clustering unlabeled data requires careful analysis and selection of appropriate features to ensure accurate grouping. Additionally, the large volume of malware samples and the variety of malicious behaviors make it difficult to identify distinct clusters and patterns. Furthermore, association analysis plays a significant role in identifying correlations between variables in malware datasets. However, due to the complexity and heterogeneity of malware behaviors, finding meaningful associations can be challenging. Unsupervised learning strategies, such as market basket analysis, face difficulties in uncovering hidden relationships and identifying relevant associations in malware data. Lastly, dimensionality reduction techniques are essential in handling the high-dimensional nature of malware datasets. By reducing the number of features or dimensions, dimensionality reduction methods aim to simplify the analysis process and improve detection accuracy. However, selecting the most relevant features and preserving critical information while reducing the dataset’s complexity is a non-trivial task.

I. Advanced Techniques for AMSI and AV bypass using encrypted PowerShell [33]

In the realm of malware detection, one of the significant challenges lies in bypassing Anti-Malware Scan Interface (AMSI) [33] and modern antivirus (AV) systems. This method, employed for AV bypass, showcases its efficacy by successfully evading AMSI and all current AV solutions on Virus Total. By employing innovative techniques, such as script compression and encryption, variable name randomization, and statement order shuffling, it achieves maximum entropy and obfuscation, making the decrypted stub highly obscure and difficult to detect. This approach not only ensures minimal overhead but often exhibits negative overhead due to efficient compression techniques. Moreover, it offers the flexibility of customization, allowing security practitioners to modify the Crypter variant as per their specific requirements. The method supports recursive layering, enabling multiple levels of encryption and compression, tested up to 500 layers.

## 4. Considerations into the Benefits and Motivations behind Open-Source Software for Malware Response and Detection

In the realm of cyber space, the need for open-source solutions has become increasingly significant. This is due to the numerous advantages they offer in terms of transparency, flexibility, and collaboration. Open-source solutions refer to software or technologies that are developed and distributed with their source code freely available to the public. In the context of security, the utilization of open-source solutions brings more benefits.

One key advantage is the transparency provided by open-source solutions. With access to the source code, security professionals can scrutinize the inner workings of the software and identify any potential vulnerabilities or weaknesses. This transparency fosters a sense of trust and confidence in the security community, as experts can independently verify the effectiveness and integrity of the solution.

Flexibility is another crucial benefit offered by open-source solutions. The nature of open source allows for customization and modification to suit specific security requirements. Security practitioners can tailor the solution to address their unique needs, integrating additional functionalities or removing unnecessary components. This adaptability ensures a more robust and tailored approach to security, enhancing overall defense against potential threats.

Another significant advantage of open-source solutions is the absence of licensing fees. Unlike proprietary software that requires the purchase of licenses, open-source solutions are freely available for use and distribution. This eliminates the upfront cost typically associated with proprietary solutions, allowing organizations to allocate their budget more efficiently.

The inherent scalability. As other price-based solutions are developed by internal contributors, the open-source have a more robust architecture that can handle increased workloads and accommodate expanding user bases. This scalability allows organizations to scale their security infrastructure seamlessly as their needs evolve over time. Through open-source platforms, security professionals can tap into the collective wisdom and expertise of a diverse community, leading to more robust and effective security measures.

Traditional tools used for system protection, such as antivirus programs employing behavioral analysis, signature-based static analysis, and heuristics, have been effective in certain situations. However, they have become inadequate in safeguarding against the increasing number and complexity of modern threats. As a result, organizations have turned to advanced security solutions, including Security Orchestration, Automation, and Response (SOAR) systems; Next-Generation Antivirus (NGAV); Endpoint Detection and Response (EDR); and Extended Detection and Response (XDR) approaches. These integrated solutions combine the traditional antivirus functionality with monitoring tools and advanced technologies such as artificial intelligence and machine learning to provide a rapid and efficient response to the most complex risks and threats. Nevertheless, implementing and utilizing these advanced security solutions can be challenging for small and medium-sized organizations, non-governmental organizations (NGOs), and government entities due to high costs and the need for specialized personnel.

Hence, the primary objective of this solution is to provide a set of accessible and free technologies that form a solid foundation for system protection in any organization or online presence. The proposed solution based on an open-source approach enables information sharing and collaboration through an Information Sharing and Analysis Center (ISAC). Leveraging collective knowledge and expertise will lead to the development of architecture and advanced protection systems against cyberattacks, benefiting any company or user seeking to adopt this solution. A scalable and configurable protection architecture has been designed and developed, incorporating multiple embedded solutions and a communication platform usable by a Security Operations Center (SOC) team.

The aim is to provide suitable utilities for monitoring, prevention, detection, and response to current and future threats, all available for free as an open-source program with a long lifespan. Secondary objectives include the development of a network monitoring program, process automation, endpoint detection and response agent deployment, as well as network security monitoring. Through this project, the goal is to offer an accessible and efficient solution for system and infrastructure protection for small and medium-sized organizations. By integrating existing technologies and developing customized components, the aim is to provide an independent solution capable of addressing the increasingly complex challenges of cybersecurity and contributing to the overall level of protection for all users and companies leveraging its capabilities.

## 5. Design and Implementation of the Security Framework for Assuring a Better Cyber Posture and Resilience

The design and implementation of the open-source security framework aimed at enhancing cyber posture and resilience. The framework encompasses various strategies, technologies, and open-source solutions to safeguard against new evolving cyber threats and ensure a resilient defense posture. Through meticulous planning and strategic implementation, this framework aims to mitigate vulnerabilities, strengthen security measures, and enable organizations to proactively respond to cyber incidents.

The goal of such a framework is to ensure that organizations have the necessary security measures in place to protect their critical assets, data, and systems. It helps in identifying vulnerabilities, implementing appropriate controls, and continuously monitoring and updating security measures to address emerging threats. Moreover, a robust security framework promotes a culture of security awareness and accountability within the organization. It enables stakeholders to understand the importance of cybersecurity and their role in maintaining a strong cyber posture.

A set of equations is proposed to perform predictive analysis using specific algorithms and models, such as ARIMA (Autoregressive Integrated Moving Average) [34], to assess and quantify relevant metrics in the field. These processes will facilitate decision-making and increase situational awareness within threat intelligence. This equation quantifies the severity of a threat by considering the criticality, impact on resources, and required reaction time.

The equation for calculating the Threat Severity Metric (*TSM*) is as follows:

TSM=α1 ∗ CA+α2 ∗ RS+α3 ∗ RT

*CA* represents the level of threat criticality;

*RS* represents the level of impacted resource severity;

*RT* represents the level of required reaction time to counter the threat;

*α*_1_, *α*_2_, *α*_3_ are the weights associated with each component.

The equation for calculating the Risk Metric (*RI*) is as follows:

RI=β1 ∗ SA+β2 ∗ CV+β3 ∗ RN

*SA* represents the threat severity;

*CV* represents the level of system or asset criticality;

*RN* represents the frequency of threat occurrence;

*β*_1_, *β*_2_, and *β*_3_ base associated each value.

The equation for calculating the Financial Impact Metric (FIM) is as follows:

γ1 ∗ A ∗ X+γ2 ∗ DT+γ3 ∗ D

*A* represents the average cost of a security breach;

*X* represents the probability of financial loss;

*DT* represents data statistics;

*D* represents costs based on a component;

γ_1_, γ_2_, and γ_3_ are the weights associated with each component.

The equation for calculating the System Performance (SPM) is as follows:

$$SP=\sum_{i=1}^{n}w_{i}\;*\;M_{i}$$

*M_i_* represents a specific metric of system performance;

*w_i_* represents the weight associated with each metric:

$$\left(w_{i}\;\geq 0\right)\;and\;(\sum_{i=1}^{n}w_{i}=1)$$

where the first condition states that the weights must be greater than or equal to zero. In the context of system performance, this means that the weights assigned to each metric should be non-negative values. The second condition specifies that the sum of all the weights should equal one. The weights must be normalized or scaled in such a way that their total sum is unified. This ensures that the weights represent a valid weighting scheme and that they cover the entire range of possible values.

*n* represents the total number of performance metrics considered.

The above metrics were used to evaluate and measure various aspects related to the performance, efficiency, financial impact, and resilience of the security system, making the solution relevant and applicable to a wide range of organizations. This includes small and medium-sized enterprises (SMEs), large corporations, government agencies, and other entities seeking to enhance their security measures and mitigate risks effectively.

The technical solution is proposed to be implemented by the following categories:

Small and medium organizations across various industries can benefit from implementing this robust security solution and whose valuable assets, intellectual property, and customer data will be protected.

Government agencies and public institutions handle sensitive information and provide critical services to citizens. The security solution could help by ensuring the confidentiality, integrity, and availability of government systems and data. It enables effective incident response and contributes to national cybersecurity initiatives.

Non-profit organizations, including charities, educational institutions, and research organizations, also need to prioritize cybersecurity. Protecting sensitive information, intellectual property, and sensitive research data is important. This solution has modules that could be applied only to those that are important.

The healthcare sector faces unique challenges due to the sensitive patient data it handles and the critical nature of its services. Security, in general, will be essential for protecting patient privacy, securing medical records, and safeguarding critical infrastructure, such as medical devices and telehealth systems.

Furthermore, the implementation of this security solution offers scalability and flexibility, making it suitable for organizations of all sizes. Its modular design allows for customization and adaptation to specific requirements, ensuring that the solution aligns with the unique needs of each organization. In addition to the categories mentioned above, other sectors, such as financial institutions, e-commerce platforms, and manufacturing companies, can also benefit from deploying this robust security solution. By bolstering their cybersecurity defenses, these sectors can mitigate risks associated with cyber threats, protect sensitive financial data, and maintain uninterrupted business operations.

Moreover, the solution’s advanced threat detection and prevention mechanisms provide proactive defense against emerging and sophisticated threats. It leverages machine-learning algorithms, anomaly detection techniques, and real-time monitoring to identify and respond to potential security incidents promptly. It is worth noting that this security solution is designed with compliance in mind. It adheres to industry standards and regulations, ensuring organizations can meet their legal and regulatory obligations. By implementing this solution, organizations can enhance their overall security posture and instill confidence among stakeholders regarding the protection of sensitive information.

In conclusion, the proposed security solution addresses the pressing cybersecurity needs of various sectors, including small to medium organizations, government agencies, non-profit organizations, and the healthcare sector. Its comprehensive features, scalability, and compliance capabilities make it an ideal choice for organizations seeking robust protection against emerging threats and the safeguarding of critical assets.

A. Proposed solution and methodology

**Figure 2:**
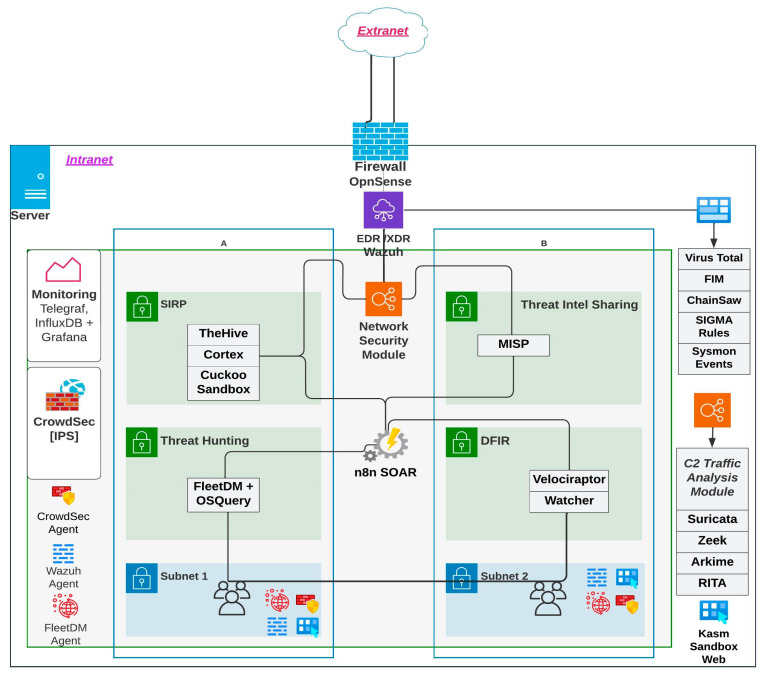
Proposed solution model diagram.

Figure 2 depicts the proposed diagram showcasing the recommended implementation. The solution is constructed by incorporating various essential technologies, including threat hunting, threat intelligence sharing, security incident and response platform, firewall, log ingestion, security information and event management, advanced firewall, and device management.

The entire system was configured using Docker technology to enable virtualization and easy deployment in any infrastructure. Apart from Cuckoo Sandbox, all components have been adapted to function within a container environment. Integrating Cuckoo Sandbox into a container presents technical challenges and is considered nearly impossible. The system implementation relied on two hardware servers, which were configured and equipped with specific modules and resources.

Regarding the primary server, it has been enriched with a range of state-of-the-art tools installed through the Docker platform using Docker-compose to ensure optimal network security and management. These systems include Wazuh, which functions as a Security Information and Event Management (SIEM) system, as well as an XDR and EDR system capable of detecting any threat. Alongside Wazuh, there are TheHive, Cortex, n_8_n, Cuckoo Sandbox, and MISP [35], all fulfilling the functionalities of a Security Incident Response Platform (SIRP). FleetDM with OsQuery serves as the threat-hunting and identification module. Telegraf, InfluxDB and Grafana serves as monitoring tool.

The secondary server has been meticulously configured to operate as a firewall, leveraging the OpnSense platform [36]—an advanced and free solution for security and network management. Additionally, it has installed CrowdSec, an innovative security solution that employs behavioral analysis to detect and prevent cyber-attacks. CrowdSec harnesses the collective power of a global network of users, gathering and analyzing security data using sophisticated machine-learning algorithms to identify suspicious patterns and malicious activities.

Additionally, the solution it embraced was the network security, digital expertise, and incident response modules, while also streamlining processes through automation using n_8_n. However, Cuckoo Sandbox [37], a crucial tool for analyzing potentially malicious files, was directly installed on the system due to the current version (2.0.7) because it does not support virtualized environments. VMCloak and VirtualBox were utilized to set up the dedicated Windows 7 machine Cuckoo Sandbox environment.

Together, the components from the presented framework that enhances detection capabilities, streamline incident response, and strengthen overall security posture will be explained. The open-source technologies benefit from continuous community contributions, ensuring ongoing innovation and staying ahead of emerging threats. The flow of the solution is presented in the figure below.

B. Problem definition in detection, defense, response

One of the main difficulties in malware detection is the ever-evolving nature of malware. Malicious actors continuously develop new techniques, obfuscation methods, and evasion tactics to evade detection by traditional security measures. As a result, security professionals must constantly adapt and improve their detection methods to stay one step ahead of these evolving threats.

Another challenge is the sheer volume of malware samples that need to be analyzed. With the proliferation of malware variants and the increasing sophistication of attack campaigns, security systems are faced with a massive influx of potential threats. Efficiently processing and analyzing this vast amount of data is a significant challenge for malware detection solutions.

Malware can manifest in various forms, including viruses, worms, Trojans, ransomware, and more. Each type of malware may exhibit unique characteristics, behaviors, and propagation methods. Therefore, accurately detecting and classifying different types of malware requires comprehensive analysis techniques and specialized detection algorithms.

Malware development techniques [34,38] employ various obfuscation techniques to disguise their malicious intent and evade detection. This includes code obfuscation, polymorphism, encryption, and other evasion tactics. These techniques make it challenging for traditional signature-based detection methods to identify and categorize malware accurately. In conclusion, the ever-evolving nature of malware, the overwhelming volume of samples, and the obfuscation techniques employed by malicious actors present significant challenges for malware detection. Addressing these challenges requires a multi-layered and adaptive approach that combines advanced analysis techniques, machine learning, and continuous monitoring. By staying vigilant and employing cutting-edge detection methods, security professionals can mitigate the risks posed by malware and safeguard their systems and data from malicious threats. To further complicate matters, malware authors frequently update their malware to evade detection. They employ zero-day exploits, social engineering tactics, and targeted attacks to bypass traditional security measures. Therefore, continuous monitoring, timely updates, and proactive threat hunting are critical components of an effective malware detection strategy.

In response to these challenges, advanced malware detection solutions utilize a combination of approaches. These include behavior-based analysis, machine-learning algorithms, heuristics, sandboxing, and threat intelligence feeds. By leveraging these techniques, security professionals can enhance their capabilities to detect and respond to emerging malware threats effectively.

C. Proposed System Architecture and Flow

**Figure 3:**
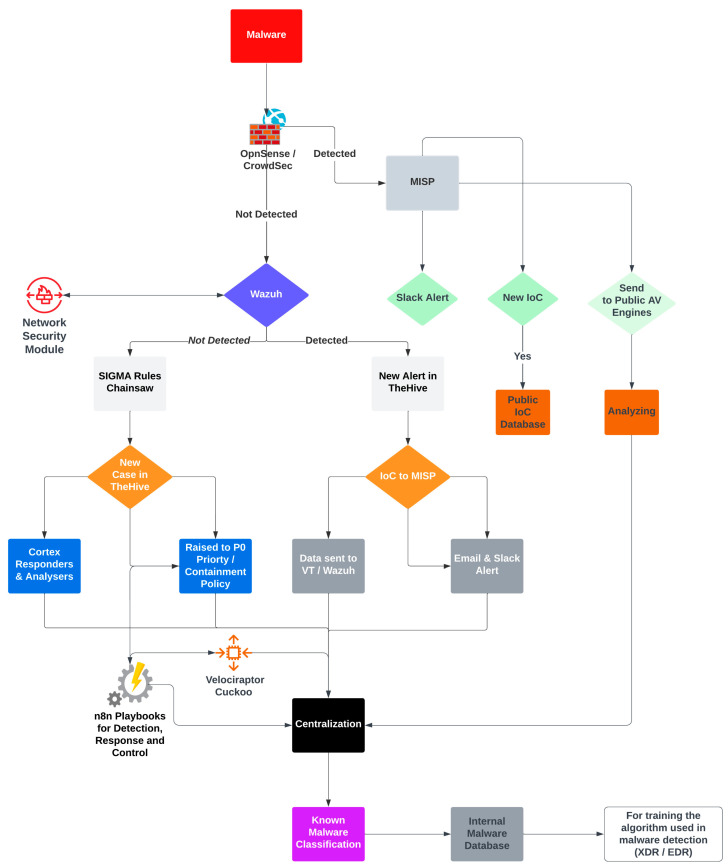
The flow of the proposed anti-malware solution.

Figure 3 describes the proposed anti-emerging threat solution that aims to provide robust protection against evolving and emerging threats, enabling organizations to proactively detect, mitigate, and respond to potential risks in a timely manner. Described below is the flow for the proposed emerging anti-threat solution related to Figure 3:

(I) Malware is delivered via email using file formats such as Microsoft Word (docx) and Excel (xlsx) utilizing attack methods such as Word’s remote template feature and Microsoft Support Diagnostic Tool (MSDT). The malware utilizes VBA macro code to execute shellcode stored in the document’s properties, which arrives as an email attachment. The attack also involves abusing macros to achieve code execution from a Word document, as well as embedding malware within Microsoft Office documents.

(II) In one scenario, the malware is discharged within the company on one of the personal computers, or it is stealthily shared as a hidden executable by an insider threat to a colleague.

(III) The traffic flow passes through the First Server, where OpnSense and CrowdSec are installed. OpnSense provides multiple functions, such as state packet inspection (SPI), anti-spoofing, deep packet inspection (DPI), and L2 to L7 traffic detection. Additionally, OpnSense includes a ClamAV plugin that can work in conjunction with the C-ICAP plugin or leverage third-party engines from well-known vendors.

OpnSense, as a network security platform, enables scanning for viruses directly on the router, preventing malicious network packets from entering the network. This approach is particularly useful for guest networks and cases where all clients may not have up-to-date antivirus programs installed. Utilizing a central antivirus system adds an extra layer of protection based on the defense-in-depth principle.

CrowdSec, integrated with OpnSense, enhances the solution by scanning and analyzing network traffic, detecting suspicious activities, sending alarms to IT personnel, dropping malicious packets, blocking malicious traffic, and performing stateful protocol analysis detection. It checks for protocol anomalies and unusual TCP segmentation and flags combination, corrupt checksums, incorrect IP fragmentation and reassembly flags, erroneous source and destination port numbers, illegal protocol commands and their usage, running protocols on non-standard ports, presence of shellcode in unexpected application protocol fields, and misuse of protocol and protocol services.

CrowdSec utilizes appliance sensors that analyze traffic and detect attacks. These sensors examine packet headers and data content, searching for patterns and behaviors indicative of malicious activity. When an attack is detected, the sensor sends alerts to the management console and can respond according to predefined policies, which may include generating alerts, logging events, resetting TCP connections, blocking traffic, scrubbing malicious packets, or dropping packets.

(IV) Wazuh serves as the central piece, functioning as a SIEM, XDR, and EDR solution. It is enhanced through various integrations, including VirusTotal, FIM, Sigma Rules, SysMon events, CrowdSec integration, and C2 IoC detect integration. The network security module relates to Wazuh and OpnSense (optional), and it is configured with modules such as C2 analysis, Suricata, Zeek, Arkime, and RITA.

(V) If the traffic is detected using OpnSense and CrowdSec with their respective modules, an IoC (Indicator of Compromise) is sent to MISP along with the relevant attack information. Slack is used for alerting, and the new IoC is shared in the IoC database. The malicious file is sent for analysis to multiple antivirus engines, such as Avast, AVG, Bitdefender, and others. The results are centralized in an internal malware database.

(VI) If the malicious file is detected, a new alert is created in TheHive platform. Details about the malicious file are sent to VirusTotal and Wazuh. An IoC is sent to MISP, and administrators are notified via email and Slack.

(VII) If the file is not detected, Cortex analyzers and responders can be utilized. Modules, such as Cuckoo Sandbox for analyzing potentially harmful files and Velociraptor for threat hunting, can also be employed. Cortex analyzers and responders are used to determine if the file is malicious or not. The collected data are centralized in a database, which serves as a known malware classification database and helps train algorithms for detecting new malware families.

D. Experimental Setup

The implementation, testing, and development of the solution required a well-designed setup comprising various infrastructure components. These components were chosen to provide all the necessary resources and ensure a robust environment. Overall, this setup provided all the required resources for the implementation, testing, and development of the solution. It encompassed servers, virtualization, storage systems, networking infrastructure, monitoring and management tools, development, and testing environments, as well as backup and disaster recovery mechanisms. This well-designed infrastructure played a crucial role in supporting the successful deployment and operation of the solution while ensuring scalability, reliability, and security.

## 6. Experimental Results and Analysis

To thoroughly investigate and test the proposed solution, it was crucial to establish a secure environment consisting of containerized applications deployed on servers. This environment encompassed not only the necessary software running on internal hosts but also enhancements to network and system resources and other associated services. Given the sophisticated nature of modern malware-targeting networks and running services, it was imperative to create an emulated environment that allowed malware to execute in a controlled manner for testing purposes, leveraging common services such as Domain Name Service (DNS) or Simple Mail Transfer Protocol (SMTP). To achieve this, the authors opted to utilize VirtualBox by Oracle in conjunction with VMCloak, ensuring a secure environment for Cuckoo Sandbox analysis. Additionally, Docker and VirtualBox were selected for test experiments and the creation of a safe environment, primarily due to the authors’ familiarity with these software solutions, although other options could have been chosen based on performance and suitability.

Malware samples were obtained from diverse sources, including specific websites dedicated to hosting malware samples and educational institutes or personal GitHub pages that provide such samples for academic purposes. The authenticity and validation of these malware samples were tested using the designed system proposed in this research. Notable online databases, such as Malware Bazaar and Malware Hash Registry, were utilized as references during the validation process. Before downloading or transferring any collections onto a system, it was vital to ensure the implementation of comprehensive containment measures within the environment. This entailed designing a solution capable of detecting and analyzing malware files and network resources, serving as a prototype for digital forensics and security. The proposed solution exhibits the ability to perform crucial processes that aid in analyzing infected files and providing alert detection and response capabilities.

## 7. Testing the Prototype

A systematic approach was developed based on existing work, providing comprehensive guidelines on how to securely install malware samples for testing purposes. To facilitate testing, a newly installed Windows 10 virtual machine was set up using VMWare. Various agents, including CrowdSec, Wazuh, FleetDM, and Velociraptor, were installed within this isolated Windows 10 environment. The prototype was initiated only when the operational solution was deemed ready to handle the proactive installation of malware samples. The selection of appropriate sample malware was a critical aspect of the testing environment. These samples were downloaded from reputable websites and other trusted sources, adhering to ethical considerations. The sources provided malware samples explicitly intended for research and investigative purposes. Prior to commencing the investigation, the following factors, based on established best practices, were considered:

(a) Ensuring the integrity and safety of the virtual environment before downloading any malware, thereby preventing the infection from spreading to the base system or other connected wired or wireless network nodes.
(b) Carefully understanding the nature and scope of the malware infection to determine the appropriate investigative tools to employ.
(c) Continuous monitoring and analysis of processes within the installed environment to gather knowledge and data on malware behavior. This information later contributed to evaluating the effectiveness of the selected tools in the development phase.
(d) Recording the system’s initial state as a reference point for later comparison with its status after malware infection.
(e) Documenting network statistics, including the IP address and subnssset mask of the system, to differentiate between authorized and unauthorized sources in the experimental results.
(f) The installed agents provided valuable insights from the perspective of the malware and collected relevant data to enhance the understanding of the malicious activity.

### Test Cases Used for Testing the Proposed Solution

(I) First case is based on AMSI Bypass technique using .NET ALI Call Hooking [39,40].

In this paper, a novel technique to bypass Microsoft’s Anti-Malware Scan Interface (AMSI) using API Call Hooking of CLR methods is presented. By leveraging this technique, the execution of malicious code on Windows systems can proceed without interference from installed antivirus solutions. This method offers advantages over other API Call Hooking approaches that target native functions, such as AMSI.dll::AmsiScanBuffer. It poses a greater challenge for the system, or Application Protection rules commonly employed in enterprise environments, and is effective against PowerShell versions _3_._0_+, including PowerShell _7_+.

Bypassing Microsoft’s Anti-Malware Scan Interface (AMSI) remains a favored tactic among cyber threat actors seeking to avoid detection. By successfully circumventing AMSI, malicious actors can disable further interference from antivirus solutions such as Windows Defender ATP, facilitating the loading of malicious or signature-based payloads. Using the API Call Hooking of .NET method, which relies on API Call Hooking of .NET methods, capitalizes on the fact that .NET methods are compiled into native machine instructions in memory, closely resembling native methods. By hooking these compiled methods, the control flow of a program can be altered to achieve the desired outcome.

Implementation steps. The following steps outline the process of the API Call Hooking for .NET method:

∘ Identifying the target method to hook;
∘ Defining a method with the same function prototype as the target method;
∘ Utilizing reflection to locate the target methods;
∘ Ensuring compilation of each method;
∘ Locating the memory addresses of the target methods;
∘ Overwriting the target methods with instructions redirecting to the designated malicious method.

The results are as follows. In the first PowerShell screen, it can be noticed that the PowerShell script for bypassing AMSI has been executed, and nothing was detected by the Windows Defender. The second screen shows that, by invoking the Mimikatz, Defender has discovered the operation.

By using the AMSI bypass PowerShell script, it becomes possible to bypass the Defender Anti-Malware Scan Interface, as evidenced in the preceding figure. Conversely, the subsequent display of PowerShell commands showcases the efficacy of AMSI in blocking the execution of the “Invoke-Mimikatz” command. This demonstrates the assertion that without utilizing the AMSI bypass script, the Defender diligently blocks any malicious endeavors.

**Figure 4:**
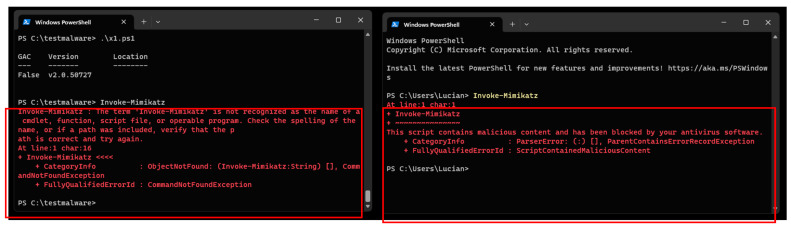
Execution of the AMSI bypass script for “Invoke-Mimikatz” command.

In Figure 4 of this study, various techniques were evaluated to bypass the Anti-Malware Scan Interface (AMSI). To conduct the experiments, a specific script, accessible at the following URL: https://github.com/pracsec/AmsiBypassHookManagedAPI (accessed on 9 June 2023) was utilized. The script allowed executing the “Invoke-Command” function within the initial Windows PowerShell session without being detected by the antivirus software. However, as depicted in the second image, when the “Invoke-Mimikatz” function was triggered, the script was caught by Microsoft Defender Anti-Virus, indicating the ability to bypass the AMSI controls. This observation serves as evidence of the potential to circumvent the standard AMSI-based security measures provided by Microsoft Defender Anti-Virus.

Upon executing the “Invoke-Mimikatz” command, as indicated in Figure 4 on the second PowerShell interface, the Windows Defender detected and intercepted the potentially malicious Mimikatz script.

A notification has been generated through TheHive platform, indicating the detection of a malicious event by Wazuh, utilizing its internal SIEM engine, specifically Filebeat. The detected event was promptly forwarded to TheHive for further analysis and response.

**Figure 5:**
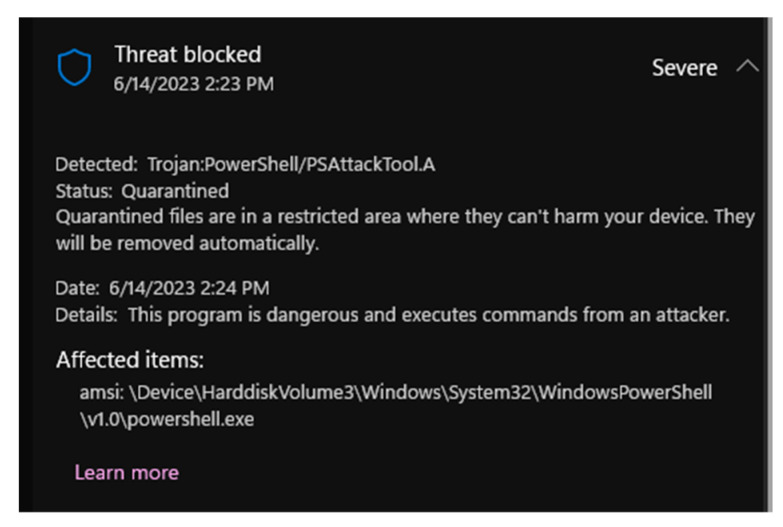
In the above image, it can be noticed that the Defender caught the Mimikatz execution (from the second list).

Figure 5 illustrates the alert generated by Wazuh, an EDR/XDR solution, and its seamless integration with TheHive platform. Upon receiving the alert, TheHive promptly initiates the creation of a new case. This integration between Wazuh and TheHive enables efficient incident response and facilitates the management of security events in a streamlined method.

**Figure 6:**
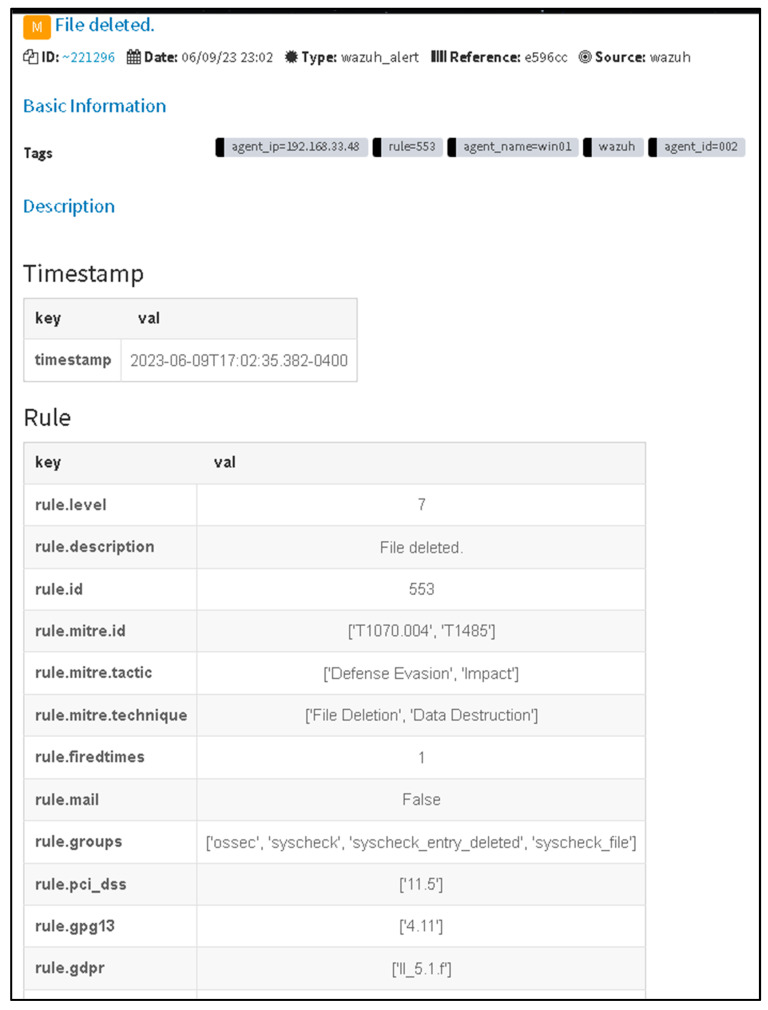
It can be noticed that the Wazuh detected the file as suspect and created a new case to TheHive.

As shown in Figure 6, the information provided illustrates the basic process of handling incidents using The Hive platform showing information related to what happens with the suspicious file that has been detected as malicious. Through the utilization of a Wazuh integration, it becomes feasible to configure Slack as a notification channel to receive alerts regarding newly detected malicious files. This integration allows for the seamless transmission of alerts from Wazuh to Slack, providing real-time updates and enhancing the organization’s ability to swiftly respond to potential security threats.

**Figure 7:**
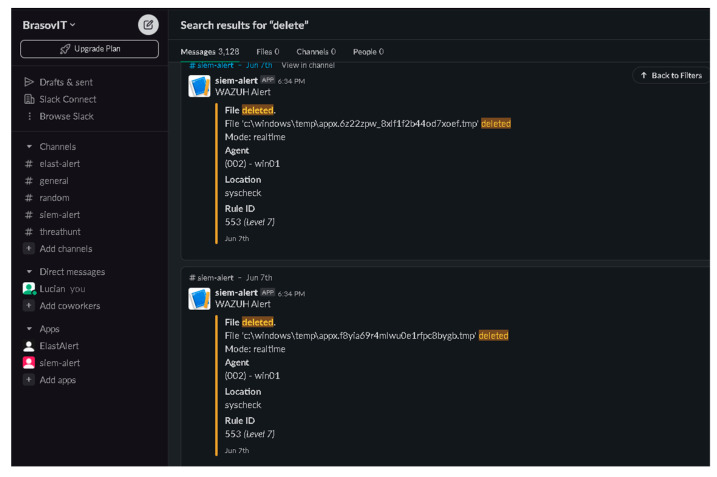
It can be noticed that Wazuh sent an alert to Slack to inform the administrators.

As illustrated in Figure 7, a notable observation arises from the incident flow, wherein Wazuh, the security monitoring platform, effectively generated an alert and subsequently transmitted it to Slack platform via web hooks. A Wazuh rule can be configured to connect with the Cuckoo Sandbox. This rule will trigger whenever a file is identified as malicious by Wazuh. Until detection, the rule using the MD5 and SHA256 information from the file hashes will be sent to Cuckoo Sandbox for comprehensive scanning and analysis.

**Figure 8:**
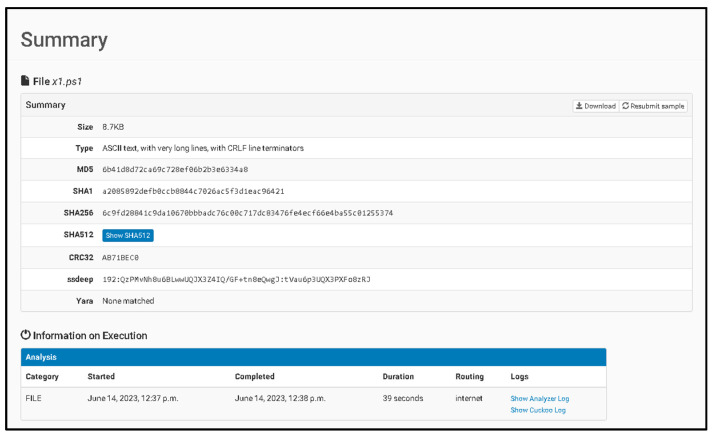
It can be noticed that the Cuckoo Sandbox received the file using the n_8_n automation.

Figure 8 exemplifies the capability of the Cuckoo Sandbox to analyze a malicious PowerShell script (accessible at the following URL: https://github.com/PowerShellMafia/PowerSploit/blob/master/Exfiltration/Invoke-Mimikatz.ps1 (accessed on 10 June 2023) with the objective of identifying potential instances of executing a harmful PowerShell script. The Cuckoo Sandbox serves as a powerful tool for examining and detecting the execution of malicious PowerShell scripts, contributing to enhanced threat intelligence, and bolstering the overall security posture of the system.

**Figure 9:**
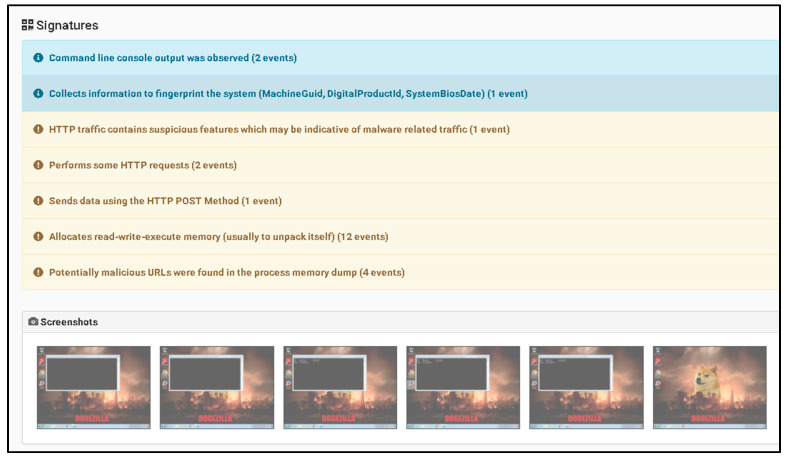
The Cuckoo file analysis shows that the file is detected to be malicious.

As shown in Figure 9, the information provided illustrates the collected information from the Cuckoo Sandbox platform which shows information related from a previous scanned malicious script.

**Figure 10:**
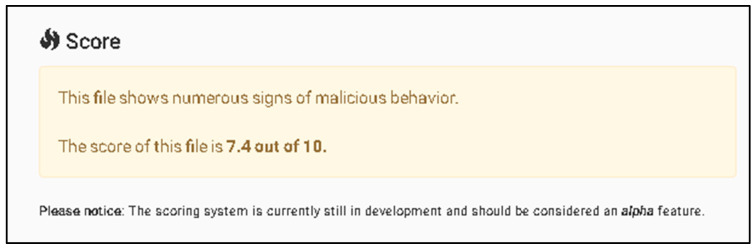
Score received from Cuckoo Sandbox.

As shown in Figure 10, the information provided illustrates the score assigned by the Cuckoo Sandbox system to the potential malicious file, which is high (7.4) from 10, where 10 is the higher score. The full log from the Wazuh system demonstrates that the malicious payload has been deleted. Using multiple verification techniques and active answers against possible new threats provides a fast incident response against all emerging threats.

**Figure 11:**
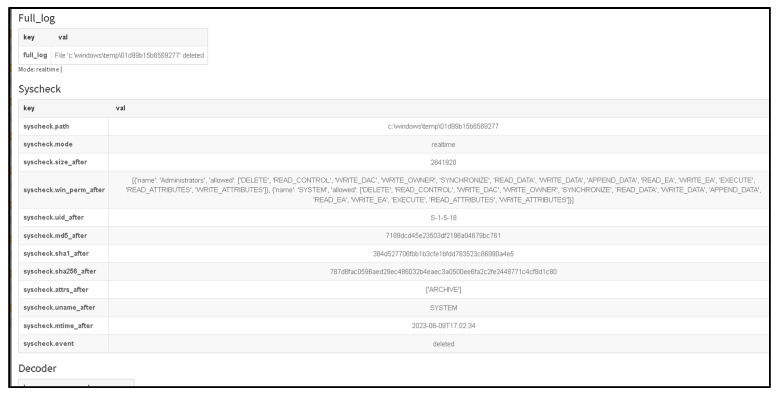
Information received from Wazuh related to malicious PowerShell script removal.

Figure 11 highlights the significant observation that Wazuh successfully removed the “malicious test script” from the desktop. This successful action demonstrates the effectiveness of the proposed solution in promptly addressing and eliminating potential security threats. The ability of Wazuh to detect and remove the identified problem further substantiates the robustness of the implemented solution in mitigating risks and maintaining a secure environment.

(II) Second case is based on disabling all anti-virus protection and firewalls from Windows 10 and running a Qbot/Qakbot-obfuscated JavaScript malware [40].

For more advanced testing purposes, the Defender (Windows Security) has been disabled.

**Figure 12:**
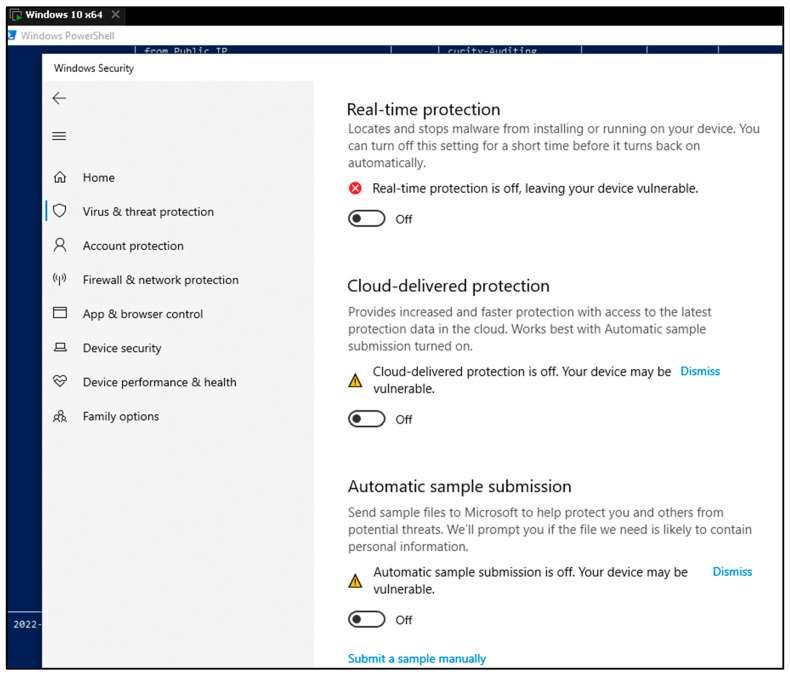
Disabled Windows Defender and security in the test environment.

As noticed in the Figure 12, where the Windows Defender were disabled and after it was possible to execute the malicious JavaScript payload obtained from the URL “http://3dg.com.br/hesqlgceuh/rentfree.zip (accessed on 12 June 2023)”, a substantial amount of log noise was generated. This log noise indicates a high volume of log entries and events generated because of the payload’s execution. A VirusTotal (VT) module with Sigma detection rules, a Chainsaw module, and an active incident response mechanism for handling unknown incidents was used.

**Figure 13:**
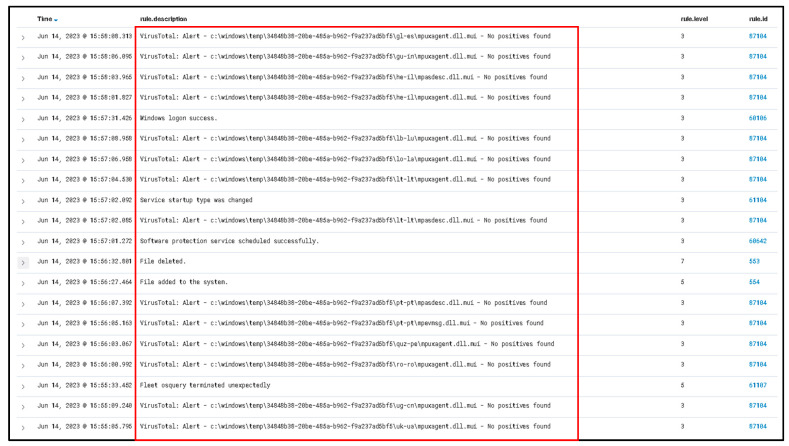
It can be noticed that the malware has been removed by using Chainsaw with SIGMA Rules and running Cuckoo Sandbox against the received file.

Figure 13 presents the dashboard of the Wazuh platform, showcasing the capability of the proposed solution to detect and identify the version of Qakbot. The Wazuh platform provides an intuitive interface that displays relevant information and insights about the identified threat. In this case, the solution successfully determined the specific version of Qakbot, contributing to improved situational awareness and enabling appropriate response measures to be taken to address the identified security issue. The dashboard view in Figure 13 demonstrates the effectiveness of the proposed solution in uncovering and managing the presence of Qakbot within the system.

**Figure 14:**
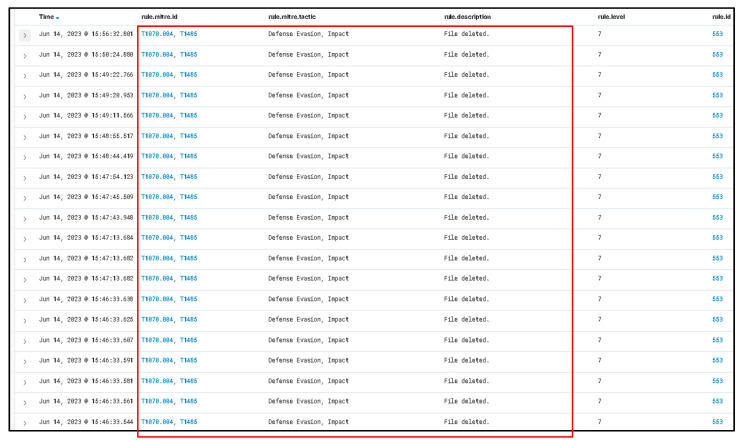
It can be noticed that all the generated artifacts from the Qakbot malware have been deleted.

Figure 14 presents the MITRE ID, tactic, and corresponding description associated with the specific files that Qakbot attempted to download and execute on the internal Windows system. MITRE, in collaboration with the cybersecurity community, has developed the MITRE ATT&CK framework, which serves as a comprehensive knowledge base of adversary tactics, techniques, and procedures (TTPs).

Each entry in Figure 14 represents a distinct TTP utilized by Qakbot, providing valuable insights into the attacker’s behavior and intentions. The MITRE ID serves as a unique identifier for the specific TTP, enabling standardized referencing and information sharing within the cybersecurity community. The tactic column categorizes the TTP based on the different stages of a cyber-attack, such as initial access, execution, persistence, and exfiltration, among others. Finally, the description column provides a detailed explanation of the TTP, outlining the specific actions or methods employed by Qakbot.

Analyzing the MITRE ID, tactic, and description in Figure 14 empowers security practitioners and researchers to gain a deeper understanding of Qakbot’s modus operandi. This information aids in the development of effective detection and prevention strategies, as well as enhancing incident response capabilities. By mapping observed TTPs to the MITRE ATT&CK framework, security professionals can better align their defenses, proactively identify potential weaknesses, and mitigate future threats posed by Qakbot or similar adversaries.

The proposed solution demonstrates a shorter time to detect (TTD) of 1.37 s from the initial point of executing a potential malicious file (T0). This signifies that the solution promptly identifies and categorizes the file as a threat within a second and a half. The detection speed is crucial as it allows for immediate incident response, enabling security teams to swiftly initiate necessary measures to contain and neutralize the threat before it can cause significant damage (Table 1).

In comparison, Windows Defender, a widely used antivirus software, exhibits a TTD of 5.66 s. This delay could provide an extended window of opportunity for the threat to propagate and inflict other malicious attacks.

Alternatively, AVG Ultimate, another security solution, offers a TTD of 2.10 s. It still demonstrates a relatively rapid response to potential threats. Additionally, AVG Ultimate comes at a cost of EUR 79.99 per year for 10 endpoints, indicating a financial investment required to access its protective capabilities.

The proposed solution’s cost is not explicitly mentioned in the given information, suggesting that it may vary or require further clarification. Further evaluation is needed for its overall cost-effectiveness, considering the solution’s TTD, additional features, customer support, and compatibility with existing infrastructure.

The cost of a security solution should be assessed alongside its performance, features, and support to determine the most suitable option that aligns with an organization’s budget and security requirements. The proposed solution is a very good option for SME’s which do not plan a budget for the current year and is a good option when a security infrastructure has collapsed (due to lack of poor acquisition management).

## 8. Conclusions

The utilization of open-source software for protection against new emerging threats presents a promising avenue in the field of cybersecurity. The inherent nature of open-source solutions, characterized by transparency, collaboration, and community-driven development, offers distinct advantages in addressing the ever-evolving threat landscape. By harnessing the collective intelligence and expertise of a diverse community, open-source software enables rapid identification and mitigation of emerging threats. The collaborative model promotes continuous improvement and adaptation, facilitating the development of robust and resilient security solutions.

Open-source software encourages scrutiny and peer review, promoting transparency and accountability. This transparency not only enhances the detection of vulnerabilities but also fosters trust among users and instills confidence in the security measures employed. Moreover, open-source solutions are often more cost-effective, as they eliminate the need for licensing fees and provide flexibility in customization and integration with existing security infrastructure. This accessibility makes advanced security capabilities accessible to a broader user base, including organizations with limited resources. However, it is important to note that open-source software is not without its challenges. Proper implementation, configuration, and ongoing maintenance are essential to ensure the effectiveness and security of these solutions. Additionally, organizations should carefully evaluate the reputation, community support, and maturity of the open-source projects they adopt to mitigate potential risks.

In conclusion, open-source software presents a compelling option for protection against new emerging threats. Its collaborative nature, transparency, and cost-effectiveness empower organizations to stay ahead of evolving security challenges while fostering innovation and community-driven development. When implemented and maintained with diligence and best practices, open-source solutions can serve as valuable tools in fortifying defenses against emerging threats in the dynamic landscape of cybersecurity. This solution/implementation is based on an open-source proposal for companies that could not afford a complete security solution.

## Figures and Tables

**Table 1:** Comparable costs between proposed solution and other enterprise software.

Solution Used	Method Used for Testing the Capabilities	TTD (Time to Detect)	Suitable for Internal Threats	Costs
Proposed solution	A. Advanced Techniques for AMSI and AV bypass using encrypted PowerShell and B. Qakbot	1.37 s	Yes	0
Windows Defender	A. Advanced Techniques for AMSI and AV bypass using encrypted PowerShell and B. Qakbot	5.66 s	No	0
AVG Ultimate	A. Advanced Techniques for AMSI and AV bypass using encrypted PowerShell and B. Qakbot	2.10 s	Yes	EUR 79.99/year

TTD = The time required to detect a potential malicious file from the initial point of execution, commonly denoted as T0, which shows the duration between the instant a malicious file is executed and the point at which it is successfully identified as a threat by the proposed solution. Cost = the general cost of the proposed solution.

## Data Availability

Not applicable.

## Appendix A

As shown in Figure A1, the information provided illustrates what is inside the directories.

As shown in Figure A2, the information provided illustrates the total number of directories and files which could be found to the related server.

As can observed in the Figure A3, the first server who could be seen in the figure is where OpnSense and CrowdSec configured. The below server is there where the prescretive security solution is installed and configured. Those represents the security stack of the solution.

Configuration of the Hardware Stack:

(I) Server I (OpnSense and CrowdSec)

Processor: i5 8600 @ 3.10 GHz

Ram: 16 GB DDR 4 @ 2666 MHz

SSD Space: 512 GB

RJ45 Adapter: three (two standard + one USB 3.0)

Operating System: FreeBSD 13.1

Estimated Cost: USD 110

(II) Server II (Security Stack)

Processor: i5 8500T @ 3.50 GHz

Ram: 32 GB DDR 4 @ 2666 MHz

SSD Space: 512 GB

RJ45 Adapter: three (two standard + one USB 3.0)

Operating System: Debian 11

Estimated Cost: USD 410
